# Optimizing Numeric Pain Rating Scale administration for children: The effects of verbal anchor phrases

**DOI:** 10.1080/24740527.2017.1398587

**Published:** 2017-12-05

**Authors:** Megan A. Young, Bernie Carter, Carl L. von Baeyer

**Affiliations:** a Department of Psychiatry, Max Rady College of Medicine, University of Manitoba, Winnipeg, Manitoba, Canada; b Clinical Nursing Research Unit, Alder Hey Children’s National Health Service Foundation Trust, Liverpool, UK; c Edge Hill University, Ormskirk, Lancashire, UK; d Department of Clinical Health Psychology and Department of Pediatrics and Child Health, Faculty of Health and Social Care, Max Rady College of Medicine, University of Manitoba, Winnipeg, Manitoba, Canada

**Keywords:** verbal anchors, Numeric Rating Scale, child, pediatric, pain, NRS, VNRS

## Abstract

**Background**: The 0–10 Verbal Numeric Rating Scale (VNRS) is commonly used to obtain self-reports of pain intensity in school-age children, but there is no standard verbal descriptor to define the most severe pain.

**Aims**: The aim of this study was to determine how verbal anchor phrases defining 10/10 on the VNRS are associated with children’s reports of pain.

**Methods and Results: Study 1**. Children (*N *= 131, age 6–11) rated hypothetical pain vignettes using six anchor phrases; scores were compared with criterion ratings. Though expected effects of age and vignette were found, no effects were found for variations in anchors. **Study 2**. Pediatric nurses (*N *= 102) were asked how they would instruct a child to use the VNRS. Common themes of “the worst hurt you could ever imagine” and “the worst hurt you have ever had” to define 10/10 were identified. **Study 3**. Children’s hospital patients (*N *= 27, age 8–14) rated pain from a routine injection using four versions of the VNRS. Differences in ratings ranging from one to seven points on the scale occurred in the scores of 70% of children when the top anchor phrase was changed. Common themes in children’s descriptions of 10/10 pain intensity were “hurts really bad” and “hurts very much.”

**Discussion**: This research supports attention to the details of instructions that health care professionals use when administering the VNRS. Use of the anchor phrase “the worst hurt you could ever imagine” is recommended for English-speaking, school-age children. Details of administration of the VNRS should be standardized and documented in research reports and in clinical use.

## RÉSUMÉ

**Contexte**: L’échelle numérique verbale (ENV) de 0 à 10 est fréquemment utilisée pour l’auto-évaluation de l’intensité de la douleur chez les enfants d’âge scolaire, mais il n’existe pas de descripteur verbal pour définir la douleur la plus aiguë.

**But**: Déterminer de quelle manière les énoncés définissant 10/10 sur l’ENV sont associés à la douleur rapportée par les enfants.

**Méthodes et résultats: Étude 1**. Des enfants (*N* = 131, âgés de 6 à 11 ans) ont évalué des vignettes représentant une douleur hypothétique à l’aide de six énoncés; les scores obtenus ont été comparés aux évaluations de référence. Bien que les effets attendus en ce qui concerne l’âge et la vignette aient été observés, aucun effet n’a été observé pour les variations dans les énoncés. **Étude 2**. On a demandé à des infirmières pédiatriques (*N* = 102) de quelle manière elles enseigneraient à un enfant à utiliser l’ENV. Les thèmes communs de « la pire douleur que tu puisses imaginer » et « la pire douleur que tu aies ressentie » pour définir 10/10 ont été identifiés. **Étude 3**. Les patients d’un hôpital pour enfants (*N* = 27, âgés de 8 à 14 ans) ont évalué la douleur d’une injection de routine à l’aide de quatre versions de l’ENV. Des différences dans l’évaluation allant d’un à sept points sur l’échelle sont apparues dans les scores de 70 % des enfants lorsque l’énoncé relatif à la douleur la plus élevée a été modifié. Les thèmes communs dans les descriptions des enfants en ce qui concerne l’intensité de douleur 10/10 étaient « hurts really bad » (fait vraiment mal) and « hurts very much » (fait très mal).

**Discussion**: Cette étude démontre l’importance de porter attention aux détails dans les instructions que les professionnels de la santé utilisent lorsqu’ils ont recours à l’ENV. L’utilisation de l’énoncé « the worst hurt you could ever imagine » (la pire douleur que tu puisses imaginer) est recommandée pour les enfants d’âge scolaire anglophones. Les détails concernant l’utilisation de l’ENV devraient être uniformisés et documentés dans les rapports de recherche ainsi que dans le cadre de leur usage clinique.

## Introduction

Self-report, when available, is widely regarded as the primary source for assessment of pain severity, to be considered in conjunction with behavioral observation and knowledge of the clinical context.^1^ Children’s ability to provide self-report of pain intensity is influenced by their level of cognitive development, as well as by the scales used and children’s experience with them. Scales widely employed for self-report of pain intensity in children include the Faces Pain Scale–Revised, the Wong-Baker FACES Pain Rating Scale, the Oucher, various visual analog scales, and verbal numeric scales.^2,3^ The 0–10 Verbal Numeric Rating Scale (also known as the NRS, NRS-11, or VNRS) is the tool most commonly used to obtain self-reports of pain intensity in school-age children, adolescents, and adults.^4,5^ A recent systematic review summarizes 16 studies supporting the utility and validity of the VNRS for most children older than 7 years, with four studies including younger participants from age 6 or 7 and above.^6^

Language comprehension and vocabulary may be influential in determining children’s capacity to use a pain scale. Parents of North American young children report that their children commonly use words such as *hurt, ouch*, and *ow* rather than the word *pain*.^7^ Thus, the word hurt rather than pain within the top and bottom anchor phrases is recommended for scales used with children. This discussion includes numerical, visual analog, faces, and other pain scale formats.

The lowest value for pain intensity on pain scales is consistently defined as *no pain* or *no hurt*. However, a wide variety of phrases are commonly used to define the meaning of the maximal (10/10) anchor of the VNRS and other pain scales, as shown in Table 1, as well as in a systematic review comparing numerical scales for adults.^12^ These descriptions can significantly alter how children use a scale for self-report of pain,^13^ a phenomenon known as the *anchor effect*. Anchor effects, described as early as 1899, occur when a judgment is influenced by its context. For example, exposing subjects to an irrelevant large number will increase their subsequent estimates of a quantity, compared with prior presentation of an irrelevant small number or no number. Similar effects occur with verbal phrases used to define rating scales. A review of empirical and theoretical literature on anchor effects is available.^14^

**Table 1.** Verbal expressions used to describe 10/10 pain on the VNRS within each study and in pilot work.

Verbal expression (with source reference)	Study 1	Study 2^a^	Study 3
1. Worst pain (hurt)^b^ you could ever imagine^8^	Yes	Yes	Yes
2. Pain (hurt) as bad as breaking your arm^9^	Yes		Yes
3. Most pain (hurt)^9c^			
4. Very much pain (hurt)^10^			Yes
5. Worst pain (hurt) you have ever had^9^		Yes	
6. Pain (hurt) as bad as it could be^11^	Yes		
7. Most pain (hurt) possible^9^	Yes		Yes

^a^In Study 2, indicated anchors were those most frequently reported as used in clinical practice by pediatric nurses.

^b^The word hurt (rather than pain) was consistently employed when the scales were used with children in the current studies.

^c^In pilot work, anchor phrase 3 performed poorly and was not used in the three studies reported here.

VNRS = Verbal Numeric Rating Scale.

Verbal anchor phrases vary in clarity, concreteness, and severity. For example, *very much hurt* is less clear and less severe than *the worst hurt you could ever imagine*. Chambers and Craig showed that calibration of a self-report scale was affected by the severity of the anchors used: children consistently rated their pain higher on a scale with a smiling face (less severe anchor) serving as the bottom anchor.^13^ Moreover, highly severe top anchors may serve to reduce ceiling effects (ratings near the top of the scale), meaning that children may generally rate their pain lower on a scale with a highly severe top anchor.^15^

The overall purpose of the studies was to determine how verbal expressions used as pain scale anchors may be associated with the calibration and comprehension of a VNRS administered to children. We are unaware of any previous studies of anchor characteristics and effects within this context. The first study assessed the association of selected anchor phrases with schoolchildren’s ratings of hypothetical pain events. The second study determined what anchor phrases are used by pediatric nurses. Finally, the purpose of the third study was to assess the association of selected anchor phrases with pain intensity as rated by children undergoing a painful procedure.

## Method and results

To assess the severity, clarity, and concreteness of a set of proposed verbal anchors, pilot work was carried out with university students as participants (*N *= 98, age range 17–39 years, *M *= 19.2 years).^16,17^ Results of the pilot study are presented in supplemental online Table 1. This pilot work showed that *worst pain or hurt imaginable* was ranked highest on severity and clarity, whereas it received a moderate ranking on concreteness. For subsequent use with children, this anchor phrase was simplified to *worst hurt you could ever imagine*.

Following this pilot testing, three studies were conducted, each described separately below. Verbal anchor phrases employed in the three studies are shown in Table 1, with references to examples of publications reporting each phrase. The anchor *worst pain you have ever had*, although reported elsewhere, was not used in the present studies for two reasons: it was rated as less severe in pilot work, and its applicability in clinical practice is limited by children’s previous experience of severe pain. If the worst pain a child has previously experienced is less severe than the pain he or she is presently reporting, the scale will not logically allow any rating less than 10/10.

For Study 1, carried out in Saskatchewan, Canada, ethics approval was obtained from the institutional review board of the University of Saskatchewan. For Studies 2 and 3, carried out in England, ethics approval was obtained from the NHS Research Ethics Service (14/NW/0163). Informed consent was obtained from adult participants, and informed parental consent and child assent were obtained for child participants.

### Study 1: Relationship of VNRS anchors with pain intensity ratings for hypothetical pain events

#### Method

Standardized hypothetical pain scenarios were used within an interview to rate the accuracy and variability of VNRS scores of pain intensity. In order to reduce the duration and redundancy of the interview, counterbalanced numbers of participants from each school grade in each school that participated in the study were allocated to one of two conditions, each using anchor phrases selected on the basis of Study 1 results. This ensured that similar numbers of children of each age category from each catchment area were exposed to the two sets of three anchors. In both conditions, the vignettes and the three versions of the scale were presented in random order. In one condition, they gave a rating of the pain intensity of four events using a VNRS with one set of three top anchors (*very much hurt, hurt as bad as it could be, most hurt possible*). In the other condition, they rated the same four events using the other set of three anchors (*hurt as bad as breaking your arm, worst hurt you could ever imagine, most hurt possible*). The participants were given a gift of a pencil or sticker for their contribution to the study.

Hypothetical pain vignettes were presented using items selected from the Charleston Pediatric Pain Pictures (CPPP), a series of pictures and accompanying text that have been extensively used in previous studies.^18^ The four scenarios depicted experiences of no pain, minimal pain, moderate pain, and severe pain; see Table 2. The vignettes were read aloud to each child, one at a time, in random order; the accompanying illustrations were shown; and ratings of pain severity were requested. Complete instructions are available from the corresponding author.

**Table 2.** Study 1: Mean pain intensity ratings by CPPP vignette (*N =* 131) aggregating across all anchors. The criterion (correct) pain intensity score for assessment of accuracy is based on pilot study results.^19^

Scenario (CPPP vignette)	Criterion score	Mean	SD
No pain (reading a book)	0	0.07	0.37
Mild pain (child pinching arm)	4	3.72	2.38
Moderate pain (bee sting)	6	6.66	2.37
Severe pain (burn hand on stove)	8	7.75	1.95

CPPP = Charleston Pediatric Pain Pictures.

#### Participants

Children, ages 6–11 (*N *= 131, mean age 8.62 years, SD = 1.54), were recruited from and interviewed at four schools in Saskatchewan, Canada. All of the children were English-speaking and all were enrolled in a regular English stream rather than French immersion school program. All English stream Grade 1–5 classrooms (*N* = 20), with an average of 25 children per class, were invited to participate.

To support a hypothesis of difference between anchors in Study 1, a minimum mean difference of 1/10 was adopted a priori based on studies of the minimum clinically significant difference in pain intensity scores.^4^ The tests of the hypotheses that mean pain scores and mean error scores anchors for the vignettes differed across anchors were two within-subjects analyses of variance, one for each set of three anchors, with the anchors as categorical independent variables and pain intensity scores and error scores as parametric dependent variables.

Severity ratings for each vignette were transformed to error scores, namely, the difference between the child’s rating and the criterion correct rating based on actual average ratings by older children and adults in pilot research.^20^ For example, if a child rated a vignette as 9/10 and the criterion rating was 4/10, the error score was 5.

#### Results

In preliminary analysis, as expected, the pain ratings strongly discriminated between the four vignettes, with mean scores as shown in Table 2. Pain ratings also showed the expected effect of age,^19^ with older children giving lower pain scores overall than younger children (*r *= −0.39, *n =* 131, *P *< 0.01). The mean pain score across all vignettes and anchors for 6-year-olds was 5.52 (SD = 0.93), whereas for 11-year-olds the mean was 3.72 (SD = 1.07).

Children were able to use the VNRS to rate hypothetical pain vignettes with similar accuracy against the criterion ratings for all six anchor phrases tested. Hypothesis tests showed null effects for pain intensity scores and error scores for both sets of three anchors as described above. For the first set of three anchors, pain intensity, F(2,134) = 0.58, *P* = 0.56, eta^2^ = 0.01; error scores, *F*(2,134) = 0.55, *P* = 0.58, eta^2^ = 0.01. For the second set of three anchors, pain intensity, *F*(2,122) = 1.50, *P* = 0.23, eta^2^ = 0.02; error scores, *F*(2,124) = 2.64, *P* = 0.08, eta^2^ = 0.04.

### Study 2: Phrases used by pediatric nurses in clinical VNRS instructions and anchors

#### Method

An anonymous online survey composed of closed and open questions was used to determine the variability and patterns of how children are verbally instructed to use the VNRS for self-report of pain intensity by nurses working in the United Kingdom. The survey included six open-ended questions that allowed the participants to describe how they currently instruct a child (age 6 to 12 years) to use a VNRS to report pain intensity and to describe the verbal expressions they were using to define 0 and 10. In the planning stage of the survey it became apparent that some nurses in the UK verbalized 0 as *zero* and some as *nought*. It was clear that this was worth considering within the survey.

#### Participants

Two key e-mail lists were utilized in order to gain a breadth of response from registered children’s nurses in the UK with a range of experience in assessing and managing children’s pain. List 1 targeted health professionals (approximately 250) active within the field of children’s pain and who would typically be working within a paediatric pain team; of these, approximately 60 were children’s nurses. List 2 encompassed children’s nurses (approximately 1000) working in a variety of ward and community settings and with a diverse range of experience of pain assessment; it represented the population of nurses who would be typically involved in undertaking pain assessment on a regular basis.

#### Results

One hundred and six individuals participated in the survey; of these, 34 (32%) indicated that they currently work within a specialist nurse pain team and 62 (59%) of the nurses reported three or more years of experience working with children. Three individuals who accessed the survey were excluded from study analysis because they were not registered children’s nurses. The respondents were broadly typical of those nurses on each of the e-mail lists. Seventy-three nurses completed all of the survey questions.

The nurses gave a large variety of answers; none were exactly identical. Commonly, children were asked whether they had any pain at the moment and how they were feeling before asked to rate their pain. Seven nurses out of 74 that explained how they would ask a child to report his or her pain on a pain scale reported using a 1–10 scale rather than a 0–10 scale to rate pain intensity. In terms of child-friendly use of substitute words for pain—that is, hurt—64 of these nurses used the word pain alone in their explanation of the scale, 25 nurses used the word hurt only, and 10 nurses used the word *sore* only in substitution of pain to children. Twenty-seven nurses used a combination of the words hurt and/or sore and pain to explain the scale.

**Figure 1.**
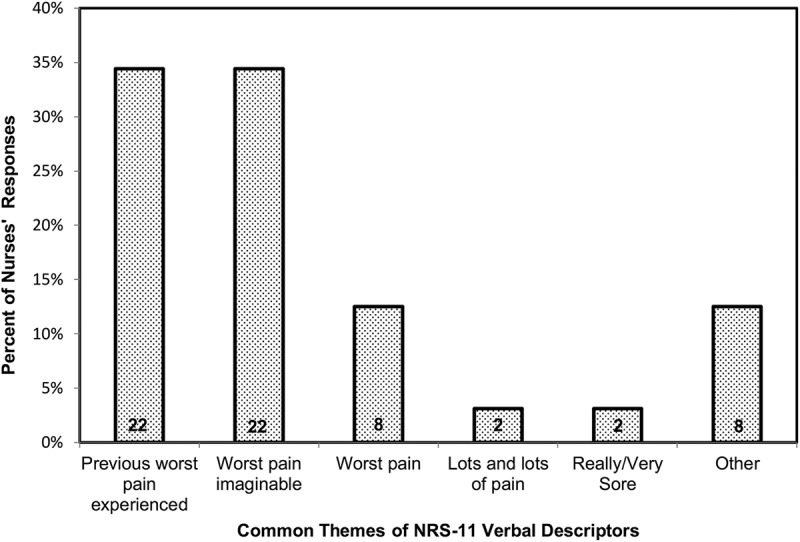
Categories of the common themes in verbal expressions used as top anchors for the VNRS by pediatric nurses in Study 2. The label of each theme is a paraphrased version of the exact wording used by nurses. Numbers inside columns show frequency for each category. All verbal expressions that held no common language are included in the “other” category.

When asked to describe the verbal explanation they use for 10/10 pain, there were some common themes in the way the questions were asked. Of the 69 who responded, 22 asked the children to think about 10/10 as the most or worst pain (hurt) they had ever previously experienced. Twenty-two others asked the children to consider 10/10 the worst pain (hurt) they could ever imagine or a very similarly worded phrase coded the same as long as they included variations of both the words *worse* and *imagine*. Other descriptions of pain that was 10/10 were worse or worst pain (*N = *8); “lots and lots of pain” (*N = *2); “really, really sore” (*N = *1) and or “very, very sore” (*N = *1). Each of the eight remaining verbal expressions were unique and each was described by one nurse only; see Figure 1.

The nurses demonstrated a great deal of variability in the exact words used to instruct a child to use the VNRS to rate pain intensity. However, the terms used in the UK were generally consistent with North American pain language, with the exception of the word nought, meaning zero, used by 26 of the 73 nurses. Nought is not commonly used by children and is not used in North American English. As such, for the final study the word zero was used to define the no pain value of the VNRS.

### Study 3: Clinical within-subject variability of children’s pain intensity ratings

#### Method

Children were interviewed and asked to provide VNRS ratings of pain severity following a repeat injection that was part of their medical treatment for an endocrine condition. Each injection was administered into a ventrogluteal injection site that had been prepared with topical anesthetic cream and vapocoolant spray. Each child was asked immediately after the procedure to report how much it hurt on four versions of a 0–10 scale, differing on verbal expression used as the upper anchor. The four anchors were chosen based on the previous study results. VNRS instructions were as follows: “Can you please give me a number from zero to ten for how much the injection hurt if zero is no hurt, and ten is the most hurt possible.” The same instructions were then repeated three more times with the verbal phrase associated with 10/10 changed to “worst hurt you could ever imagine,” “very much hurt,” and “hurt as bad as breaking your arm.” The four versions of the scale were presented in random order. Children were then asked to explain how they would describe 10/10 pain in their own words. The children were given a certificate for their participation in the study.

#### Participants

Thirteen girls and fourteen boys, ages 8–14 (mean age = 11.0, SD = 2.3; *N *= 27), receiving either a single large-volume, subcutaneous injection or intramuscular injection at a UK tertiary children’s hospital were invited to participate in the study by an endocrinology nurse upon arrival for their appointment on the ward. All of the children invited to participate had been administered these injections previously by this nurse as part of long-term treatment for endocrine conditions. All of the children recruited were able to communicate in English. No children invited declined participation.

#### Results

The children were asked to tell the researcher how much the injection they had received hurt on the four different versions of the 0–10 scale. Each child’s rating was ranked for severity across anchor types. A repeated measures analysis of variance was used to compare mean pain intensity raw scores as a function of the four anchors. The overall effect of anchors was F(3, 22) = 2.31, *P* > 0.10. In post hoc analysis of paired differences, the maximum mean difference between individual pairs of anchors was 0.28/10, SE = 0.21, *P* > 0.20.

Study 3 also permitted a within-subjects analysis of pain score across repetitions of the question. Seventeen out of 27 children (63%) gave a different pain severity rating dependent on verbal anchor. Two of the children refused to give a rating on a scale using “hurt as bad as breaking your arm” because they had not experienced this event and felt that they could not use the scale with this specific anchor attached to it. Of the children who used all four scale versions, the range of pain intensity scores from least to most pain varied from zero to seven points on the scale dependent on the scale anchor; see Table 3.

**Table 3.** Study 3: Range of absolute differences between maximum and minimum pain score across the four anchors.^a^

Absolute difference	Frequency	Percentage
0	10	37
1	8	30
2–3	6	22
4–7	3	11
8–10	0	0
Total	27	100

^a^ An absolute difference of 0 indicates that scores were the same for all four anchors.

Within the children’s responses to the open-ended question related to their own description of 10/10 pain, several themes emerged. Thirty-two percent (*n* = 9) of the children described 10/10 as a variation of “hurts really (really) bad.” Fourteen percent used a very short description and simply stated, “it hurts.” Two children stated 10/10 pain as “hurt(s) very much.” Single descriptions of 10/10 pain included “indescribable,” “it would drive you insane,” and “scary-painful, nerve-wracking.” The majority of the children used the word hurt instead of a derivative of the word pain in their descriptions. Only four of 28 of children used the word pain, and these children ranged in age from 8 to 11 years.

## Discussion

The primary aim of this series of studies was to determine how the verbal expressions used to anchor a numerical rating scale may be associated with variations in children’s ability to use the VNRS for self-report of pain intensity. Further findings demonstrated the variability and current issues in the ways in which pain scales are currently administered in clinical settings. Certain themes in children’s own pain language when defining the most severe pain also emerged within the last study.

A verbal descriptor for 10/10 pain should be severe, concrete, and in keeping with children’s pain language.^7,13^ Pilot work suggested some differences in commonly used pain scale anchors.^16,17^ The *worst pain you could ever imagine* was rated as highly severe, clear, and concrete, whereas anchors such as *the most hurt* and *very much hurt* were rated lower on these characteristics.

In Study 1, children rated hypothetical pain picture/vignettes. As expected, severely painful vignettes were rated as more painful than no-pain and mild pain vignettes. In addition, younger children, as expected, gave higher pain ratings overall, as found in several previous studies.^19^ However, although the study was powered to detect moderate-sized within-subjects variations in the effects of scale anchors, no statistically or clinically significant effects of anchors on pain ratings were found. Possible explanations for the failure to find hypothesized differences might include the following: (1) Many children might not have paid attention to the details of the changing anchors, instead focusing on the vignettes to be rated. (2) The first time the question was asked for each vignette, it anchored subsequent responses. In other words, children tended to repeat their first rating of each vignette. To examine these possibilities would require a full between-subjects design, so that each participant would rate each vignette only once. This would require many more participants (e.g., at least 50 per anchor), so a reduction in the number of anchors to only those most used would be advantageous.

Study 2 allowed for real-world exploration of how pain scales are used by knowledgeable health care professionals who are using pain scales on a regular basis to make clinical decisions regarding pain management. The actual patterns of anchor types used with the VNRS were consistent with previous international surveys of health care professionals.^9^ The equal use of variations of *previous worst pain experienced* and *worst pain you could ever imagine* demonstrated that both highly concrete and severe anchors are used in everyday practice. This suggests that experienced nurses generally understand how to phrase the VNRS top anchor in severe and concrete terms matching children’s ability to use this scale. Again, instructions referring to previous pain experience such as *worst pain you have ever had* cause problems with children’s ability to use the VNRS, so these were not tested in the final study.

Factors that may have influenced our findings about the phrases used in reporting pain include the influence of the differing linguistic and cultural contexts of the two different countries (Canada and the UK) in which the studies were undertaken. We did not set out specifically to explore language differences between the countries; our focus was on the possible influence of the verbal descriptors used to anchor a numerical rating scale. However, some British nurses used the word nought rather than zero when talking about 0, so we included a question to explore this in the survey. With the exception of the zero/nought issue, there were no apparent differences in language and descriptors commonly used when administering the VNRS in the UK when compared to language reported to be used in North American health care settings.^7,9^ There were some commonalities in how nurses asked children to report their pain; however, there was a great deal of inconsistency in the reports of the way in which the scale was explained, particularly with respect to the verbal expressions used as the top anchor for the scale. A cause for concern was that a few nurses reported using a 1–10 scale. If, the next time the scale was administered, another nurse used a 0–10 metric and attempted to compare the numbers, it would be more difficult to discern changes in pain severity.

Many nurses (*N* = 22 of 64) asked children to compare the current pain event to previous experiences of pain. There may be considerable variability in the kind and amount of pain a school-age child may have previously experienced and that he or she could use as a point of reference. Prior pain experience may be extremely different between children. An equal number of nurses used a variation of “the worst pain you could ever imagine” to describe 10/10 pain. This is consistent with the anchors often used with adults in administering VNRS for pain severity.^8^ This phrase does not have as much potential for ceiling effects as other verbal phrases.

Recent studies have demonstrated that +1 or −1 changes in VNRS were representative of a minimally clinically significant difference in pain score.^4^ Within the third study, more than half of the children changed their rating by at least one point on the scale when the verbal descriptor used as the top anchor was changed and these varied up to seven points across anchors. This means a child may indicate mild pain on one version of the scale while indicating severe pain using a variant of the scale using a different verbal descriptor for an anchor. Furthermore, this result demonstrates the critical nature of consistency in the instructions given for the scale. A change in pain severity in either direction would indicate for clinicians a potential change in the child’s condition.^4^

## Limitations

Conclusions from these studies are limited by differences between the studies. In Study 1, children were asked to rate hypothetical rather than actual painful events. The complexities of rating hypothetical, past, and future pain have recently been reviewed.^21^ Each of these pain rating tasks presents different challenges to children’s imagination, memory, quantity estimation, interoception, and other relevant cognitive abilities. Extensive developmental change in these cognitive abilities occurs between age 5 and 10 years. In particular, there are known developmental differences in attentional self-regulation, empathy, and representational theory of mind.^21^ Younger children tend to be centered on their own immediate experience and to be less able to compare it either to the imagined experiences of others or to their own experience at other times. Thus, especially for younger children, the results of Study 1 (hypothetical pain) might be expected to map poorly onto Study 3 (actual needle pain).

Moreover, unlike a clinical assessment, each child was exposed to three different anchor phrases: it is possible that children realized that they were being asked the same question three different ways and made their answers to the second and third question consistent with their first, which would diminish any possible anchor effects. Alternatively, some children, faced with the repeated question, might have thought they got the answer wrong the first time and gave a different guess on subsequent questions.

Another limitation is the discrepancy in the ages of schoolchildren in Study 1 (6–11 years, mean 8.6) versus patients in Study 3 (8–14 years, mean 11.0). In Study 1, we targeted 6 years as the lower limit of age reported in VNRS studies,^6^ whereas in Study 3 we were limited to children who were receiving certain painful injections. The small size of the clinical sample in Study 3 (*N = *27) is a further limitation imposed by the limited number of suitable patients seen in that study’s hospital setting.

## Conclusions

Pain scores are meaningful primarily in a relative sense (ipsative or idiographic, comparing self with self), not an absolute sense (nomothetic or normative, comparing self with others). One child’s rating of a painful event cannot necessarily be compared to other children’s rating of the same event: this is why comparisons of children’s ratings across anchors were made within subjects. Although the present studies were about verbally administered scales, the characteristics of anchors and the need for standardization addressed here would presumably apply equally to written numerical scales and perhaps to visual analog and faces scales. The published faces scales each have different standard instructions, including anchor phrases.^10,11^

Self-report scales are helpful for monitoring pain intensity over time when used effectively but are not relevant without adequate patient history, observation of behavior, and knowledge of the clinical context of the pain. Pain does not occur in a vacuum, and emotional states of distress and anxiety, social context, as well as the relationship with the examiner are tied to the child’s experience of pain.^22^ When a verbal numeric scale is used appropriately, with a consistent form of instructions, it can often serve as a simple, reliable, valid, and important measurement tool for pain intensity in children to assist in making decisions about treatment.

## Recommendations

Recent research has confirmed the lack of standardized instructions for the VNRS administration and defined the benefits of identifying a standard upper anchor to allow for greater comparability of use in both research and clinical settings.^6,15^ Standardization of the instructions used with the scale would allow for more consistent administration of the scale, improving monitoring and treatment.

Pending further research, we recommend the use of *worst hurt you could ever imagine* for English-speaking school-age children. This anchor is already commonly used by experienced pediatric nurses, it is a well-established standardized top anchor for verbal numeric rating scales in adults, and most children from at least age 8 are highly capable of using this top anchor to rate both hypothetical and real painful events, with minimal risk of ceiling effects. This anchor phrase is rated as highly severe and clear. It allows the child to imagine what he or she believes would be an excruciatingly painful event and create a concrete example that he or she can personally compare his or her current pain experience with. Ideally, no matter what anchor or metric is used with a numeric rating scale, this should be documented and used consistently when pain is being monitored over time.

## Supplemental data

Supplemental data for this article can be access on the publisher’s website.
